# Epidemiology of sarcoptic mange in a geographically constrained insular red fox population

**DOI:** 10.1186/s13071-024-06330-5

**Published:** 2024-06-06

**Authors:** Christy N. Wails, Claire C. Helmke, Kathleen M. Black, Roger Ramirez-Barrios, Sarah M. Karpanty, Daniel H. Catlin, James D. Fraser

**Affiliations:** 1 Department of Fish and Wildlife Conservation, Virginia Tech, Blacksburg, VA USA; 2 Department of Biomedical Sciences and Pathobiology, Virginia Tech, Blacksburg, VA USA

**Keywords:** Barrier island, Camera trapping, Ectoparasites, Epizootic outbreak, Red fox, Remote detection, *Sarcoptes scabiei*, Sarcoptic mange, *Vulpes vulpes*, Wildlife disease

## Abstract

**Background:**

Sarcoptic mange is a skin disease caused by the contagious ectoparasite *Sarcoptes scabiei*, capable of suppressing and extirpating wild canid populations. Starting in 2015, we observed a multi-year epizootic of sarcoptic mange affecting a red fox (*Vulpes vulpes*) population on Fire Island, NY, USA. We explored the ecological factors that contributed to the spread of sarcoptic mange and characterized the epizootic in a landscape where red foxes are geographically constrained.

**Methods:**

We tested for the presence of *S. scabiei* DNA in skin samples collected from deceased red foxes with lesions visibly consistent with sarcoptic mange disease. We deployed 96–100 remote trail camera stations each year to capture red fox occurrences and used generalized linear mixed-effects models to assess the affects of red fox ecology, human and other wildlife activity, and island geography on the frequency of detecting diseased red foxes. We rated the extent of visual lesions in diseased individuals and mapped the severity and variability of the sarcoptic mange disease.

**Results:**

Skin samples that we analyzed demonstrated 99.8% similarity to *S. scabiei* sequences in GenBank. Our top-ranked model (weight = 0.94) showed that diseased red foxes were detected more frequently close to roadways, close to territories of other diseased red foxes, away from human shelters, and in areas with more mammal activity. There was no evidence that detection rates in humans and their dogs or distance to the nearest red fox den explained the detection rates of diseased red foxes. Although detected infrequently, we observed the most severe signs of sarcoptic mange at the periphery of residential villages. The spread of visual signs of the disease was approximately 7.3 ha/week in 2015 and 12.1 ha/week in 2017.

**Conclusions:**

We quantified two separate outbreaks of sarcoptic mange disease that occurred > 40 km apart and were separated by a year. Sarcoptic mange revealed an unfettered spread across the red fox population. The transmission of *S. scabiei* mites in this system was likely driven by red fox behaviors and contact between individuals, in line with previous studies. Sarcoptic mange is likely an important contributor to red fox population dynamics within barrier island systems.

**Graphical Abstract:**

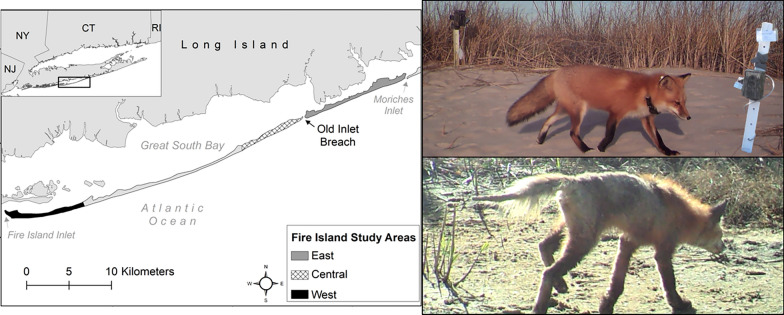

**Supplementary Information:**

The online version contains supplementary material available at 10.1186/s13071-024-06330-5.

## Background

Sarcoptic mange is a skin disease caused by the microscopic mite *Sarcoptes scabiei*, which parasitizes mammals [1, 2]. *Sarcoptes scabiei* burrows into the skin of the host, causing intense itching, skin discoloration and rashes, hair loss, and thickening of the skin [2–4]. As the mange disease progresses, the host may experience secondary infections, sepsis, and eventually death [3, 5]. High mortality rates are frequently seen following sarcoptic mange infection in wildlife, especially among wild canids [6–8]. While sarcoptic mange is an enzootic disease fully established in some species, novel outbreaks are still periodically reported [9, 10].

Over 150 mammal species from 12 orders are known to be infected with sarcoptic mange globally [5, 10, 11]. Outbreaks of infection in North America occur in a few host species, with canids being the primary host taxa [2]. *Sarcoptes scabiei* mites were likely purposefully introduced to control North American gray wolf (*Canis lupus*) and coyote (*C. latrans*) populations in the early 1900s in Montana, USA and Alberta, Canada [12, 13] and became endemic in this region with subsequent spread across the continent.

Sarcoptic mange is likely an important population driver among red foxes (*Vulpes vulpes*) [14, 15], which are highly susceptible to the disease due to their physiology, ecology, and behavior [16–18]. *Sarcoptes scabiei* mites can survive off-host for multiple days in the soil of red fox dens [19, 20], facilitating mite transmission between individuals that occupy the same dens through time [19–23]. Mortality among red foxes is typically observed 2–4 months after infection, with mortality rates ranging from 21 to 100% in infected populations [24–26], sometimes resulting in population extirpation [27]. Epizootics of sarcoptic mange are often followed by an enzootic phase where the disease can remain present in the population until another epizootic phase is reached, leading to apparent cycles of the disease [10, 13, 18].

As part of a larger study of barrier island red fox ecology [28, 29], we reconstructed the epidemiology of a sarcoptic mange epizootic in a red fox population. Our monitoring setup, developed to quantify red fox abundance and occupancy, allowed us to observe the spatio-temporal dynamics of the sarcoptic mange epizootic in a barrier island system. Unlike longer-term studies from continental systems (e.g., [15]), movements of red foxes on Fire Island are constrained geographically by the surrounding water and the narrow, elongated shape of the island, which could influence the epidemiology of sarcoptic mange. Our objectives were to (1) confirm sarcoptic mange in the study system, (2) evaluate the ecological factors that contributed to the spread of sarcoptic mange, and (3) characterize, for the first time, the spread of sarcoptic mange on a barrier island system.

## Methods

### Study site

**Fig. 1:**
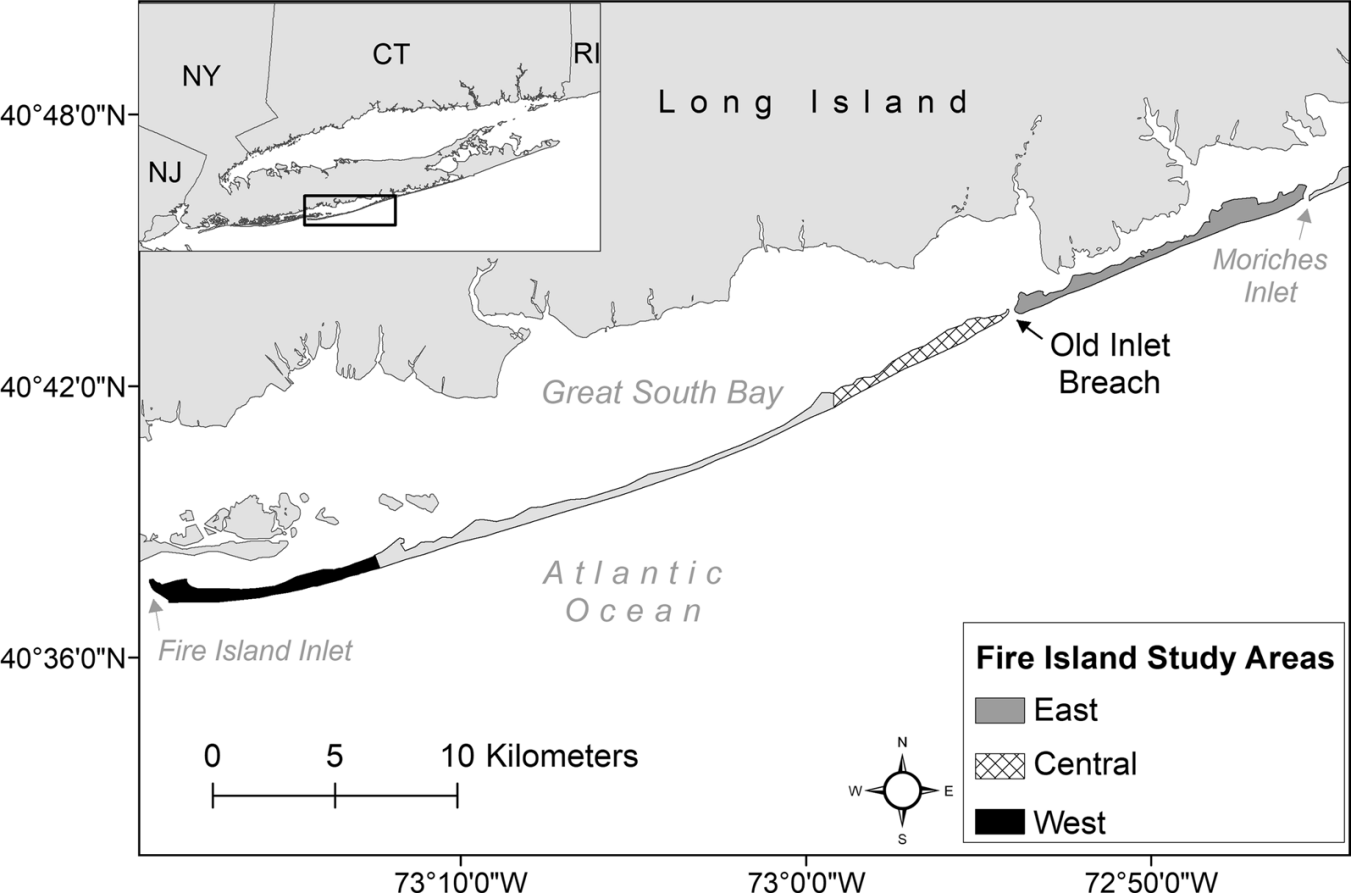
Study areas on Fire Island, NY. Research efforts were focused in three areas (from east to west): (1) east of Old Inlet breach (gray), (2) central Fire Island (hashed), and (3) west Fire Island (black)

Fire Island (40°39′ N, 73°05′ W) is an approximately 50-km-long barrier island (width: 150–1000 m) off the southern shore of Long Island, New York, bordered by Moriches and Fire Island inlets to the east and west, respectively (Fig. 1). Land use varies across the island and includes commercial districts, residential neighborhoods, county and state parks, and federally designated wilderness with ocean beach, sandy and vegetated dunes, maritime forest, saltwater marsh, and intertidal flat habitats [30]. In 2012, Hurricane Sandy breached Fire Island in two locations [31]. One of the breaches (Old Inlet) was left open, and the easternmost 10 km of the island was isolated from the 40-km western portion (Fig. 1), though bridges connected both sections of Fire Island to Long Island.

### Field methods

We monitored red foxes across the three study areas and received reports of red foxes around the interior villages from project collaborators (Fig. 1). From 2015 to 2018 we fitted up to six individuals per study area per year with Global Positioning System (GPS) tracking collars (Quantum 4000E, medium size; Telemetry Solutions, Concord, CA, USA). When we observed signs of sarcoptic mange, we wore disposable Tyvek suits (DuPont, Wilmington, DE, USA), sanitized all capture and handling equipment between captured individuals, and did not reuse traps within the same trapping period to prevent the spread of *S. scabiei* mites between red foxes. Approximately every 2 weeks, we remotely downloaded location data from GPS tracking collars. For full collaring and location monitoring methods, see [28, 29]. We recovered carcasses of deceased GPS-collared red foxes. Carcasses were individually contained and stored in a −20 °C freezer until diagnostic testing.

We used remote cameras to survey for the presence of red fox for approximately 4–5.5 months during the boreal autumn and winter of 2015–2018. Each year, we deployed 28–40 unbaited camera stations in each of the three study areas, where stations consisted of two motion-triggered cameras (Moultrie M-880c and M-880 Gen 2; Moultrie Feeders, Calera, AL, USA) mounted on T-posts. The placement of camera stations was determined using a grid overlay of each study area generated in ArcMap 10.3 (Esri Inc., Redlands, CA, USA), and camera stations were spaced 300 m (±50 m) apart [28]. The two cameras within a station were separated by 1–3 m and placed along opposite sides of game trails, clearings, or other natural travel ways. Cameras were not placed on the open beach, in deep marsh, or on pavement in order to limit water-related damage and theft. Cameras were programmed to trigger when motion was detected and to take three pictures per trigger followed by a delay of 5 s. We visited each camera station approximately every 2 weeks to replace batteries and memory cards, and clear obstructing vegetation from the field of view.

Additionally, we conducted transect surveys six times per year (three in boreal spring–summer [April/May, June, July/August], three in boreal autumn–winter [October/November, December, January/February]). We traversed 96 latitudinal transects, ranging up to 1000 m in length, across the three study areas and overlapped with the placement of camera stations. We recorded den locations and signs of red fox (e.g., tracks, scat), and opportunistically observed red foxes during transect surveys [28].

### Identifying sarcoptic mange in the system

We employed molecular methods to test for the presence of *S. scabiei* DNA from two GPS-collared red fox carcasses [32]. We collected skin samples from lesions (one sample per fox) and stored the samples in 70% ethanol until analysis. We extracted DNA using the DNeasy Blood & Tissue Kit (Qiagen, Hilden, Germany) following the manufacturer’s instructions. The second internal transcribed spacer (ITS-2) of the ribosomal RNA (rRNA) gene was amplified by polymerase chain reaction (PCR) using primers RIB-18 (GGG CTG CAG TAT CCG ATG GCT TCG T) and RIB-3 (CGG GAT CCT TCR CTC GCC GYT ACT) [33]. PCR was carried out in a 50 μl volume, using Platinum™ PCR SuperMix High Fidelity (Invitrogen, Carlsbad, CA, USA), with an initial step at 94 °C for 60 s, 35 amplification cycles at 94 °C for 60 s, 60 °C for 30 s, and 72 °C for 90 s, and a terminal extension step at 72 °C for 5 min. PCR products (c. 450 base pairs [bp]) were purified using the QIAquick PCR purification kit (Qiagen) and Sanger sequenced in both the forward and reverse directions. At least two independent PCR amplifications were performed from each sample. The resulting sequences were aligned using Geneious Prime 2021 (https://geneious.com) to construct the consensus sequences and then compared with the reference sequences from the GenBank database.

### Ecological associations with sarcoptic mange

We examined the effects of abiotic and biotic variables on the detection rates of apparently healthy (i.e., no visible signs of sarcoptic mange) and diseased red foxes (i.e., visible signs of sarcoptic mange; Appendix 1) during the sarcoptic mange outbreaks. Although we conducted camera trapping in additional years, we were primarily interested in elucidating the initial spread of sarcoptic mange. Thus, we limited our camera trapping dataset to include the eastern portion of Fire Island in 2015–2016 and the western portion of Fire Island in 2017–2018 when the sarcoptic mange outbreaks first occurred in each area.

For each camera station, we calculated detections of apparently healthy and diseased red foxes. Additionally, we recorded detections of white-tailed deer (*Odocoileus virginianus*), feral cats (*Felis catus*), northern raccoons (*Procyon lotor*), and human recreationists and their dogs (*Canis familiaris*). We calculated independent detection rates per 100 trap nights for each mammal species. We considered detections at a given camera station to be independent when species were detected > 30 min apart [34, 35] and we did not attempt to account for mammal abundance or their activity periods cf. [36].

We calculated the Euclidean distances from each camera station to the nearest red fox den that was active the previous breeding season, territory of a known diseased GPS-collared red fox, human structures (recreational vehicle [RV] parks and campgrounds, commercial districts, and residential neighborhoods), and paved roads. We digitized features from aerial imagery (15-cm resolution [37, 38]), calculated distances via the Euclidean Distance tool, and projected distance measurements to m in ArcMap 10.8 (Esri, Redlands, CA, USA). We quantified the territory of six diseased GPS-collared red foxes as the minimum convex polygons (i.e., the smallest polygon that contains all sampling points with no internal angle exceeding 180°) using all high-quality (> 3 satellites) GPS locations [28, 29].

We fit generalized linear mixed-effect models (GLMMs) with a negative binomial error distribution via the package *glmmTMB* [39] in the statistical computing environment R, ver. 4.1.3 [40]. The frequency (count) of red fox detections per station was used as the dependent variable, and we included visible signs of sarcoptic mange (apparently healthy = 0, disease = 1) as a covariate in all models, including the null. We examined the effects of human subsidies (e.g., shelters, refuse) present at park facilities and along roadways [7, 28], red fox behaviors [18, 21, 22], and activity of other mammals ([41–44], but see [2, 8]) on the detection rates of diseased red foxes. We considered eight candidate GLMMs, where visible signs of sarcoptic mange interacted with distances to the nearest human structure, nearest paved road, nearest den that was active the previous boreal spring–summer, and nearest territory of a diseased GPS-collared red fox (distances measured in meters); and detection rates of other abundant mammals (combined detections of feral cats, white-tailed deer, northern raccoons) and humans and their dogs (detection rates expressed per 100 trap nights). The global model included all two-way interactions and their constituent (additive) terms. We scaled all continuous covariates by two standard deviations to facilitate comparison of parameter estimates between continuous and unscaled binary covariates [45]. Trapping session (2015/2016 and 2017/2018) and location of camera stations were included as random effects to adjust for repeated measures. We included an offset of log-transformed trap nights in all candidate models to standardize counts. We checked for collinearity (*r* > 0.7) among all covariates and verified that the global model could meet all standard model assumptions, including spatial autocorrelation [46], via the package *DHARMa* [47]. We ranked candidate models via the Akaike information criterion [48] corrected for sample size (AIC_*c*_; [49]), and used ΔAIC_*c*_ and AIC_*c*_ weights (*ω*_*i*_) to evaluate model support [50, 51]. We selected the top-ranked model for inference when *ω*_*i*_ ≥ 0.90 [51].

### Spatio-temporal spread of sarcoptic mange

We approximated the spatial extent of the two sarcoptic mange outbreaks. We first ranked photographs of red foxes that were apparently healthy (rank 0; Appendix 1) and diseased (ranks 1–5; Appendix 1). For each week in the camera trapping session, we identified camera stations where a red fox with visible signs of sarcoptic mange was detected, and quantified the area of the minimum convex polygon for each week when sarcoptic mange was detected at ≥ 5 camera stations. Minimum convex polygons were then clipped to the outline of Fire Island to remove portions that overlapped water, using the package *adehabitatHR* [52]. We then visualized the severity (maximum mange rank per camera station; Appendix 1) and variability (interquartile range of mange ranks per camera station) in sarcoptic mange signs observed from camera trap images.

## Results

### Timeline of visible signs of sarcoptic mange

**Fig. 2:**
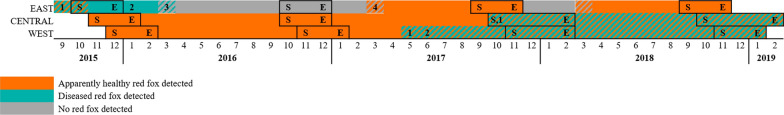
Timeline of red fox (*Vulpes vulpes*) monitoring activity and observations of apparently healthy (orange) and diseased (turquoise) red foxes during each month of the study period across the three study areas at Fire Island (top to bottom corresponds to the east to west longitudinal gradient). Camera trapping periods are identified with bold boxes and indicate start (S) and end (E) months. 1 = diseased red fox first observed at the site, 2 = first known death of a diseased red fox, 3 = extirpation of red foxes, 4 = return of red foxes. Hashing illustrates months where both apparently healthy and diseased red foxes were seen, and periods when red foxes were extirpated and later returned

In September 2015, we first observed signs of sarcoptic mange in the red fox subpopulation east of the Old Inlet breach (Fig. 2). By March 2016, red foxes were extirpated east of the Old Inlet breach and were not detected there again until March 2017. We did not observe signs of sarcoptic mange at the westernmost portion of Fire Island until May 2017, when a second outbreak of sarcoptic mange occurred at the west and central study areas. We continued to observe infected red foxes as the entire Fire Island population declined through February 2019. Excluding one instance of a northern raccoon with mange-like hair loss at the base of its tail, the visible signs of sarcoptic mange were limited to red fox.

### Identifying sarcoptic mange in the system

Seven of the 36 (19.4%) GPS-collared red foxes were later found deceased with visible signs of sarcoptic mange. Sequences from the two collected red foxes had 100% similarity, and the consensus sequence we obtained had 99.8% similarity to *S. scabiei* isolate Fox23c (National Center for Biotechnology Information [NCBI] Accession AF387730.1), with a single nucleotide variation at position 362. The sequence generated also showed > 99.5% similarity to *S. scabiei* sequences AF387717.1 and AF387727.1.

### Ecological associations with sarcoptic mange

**Fig. 3:**
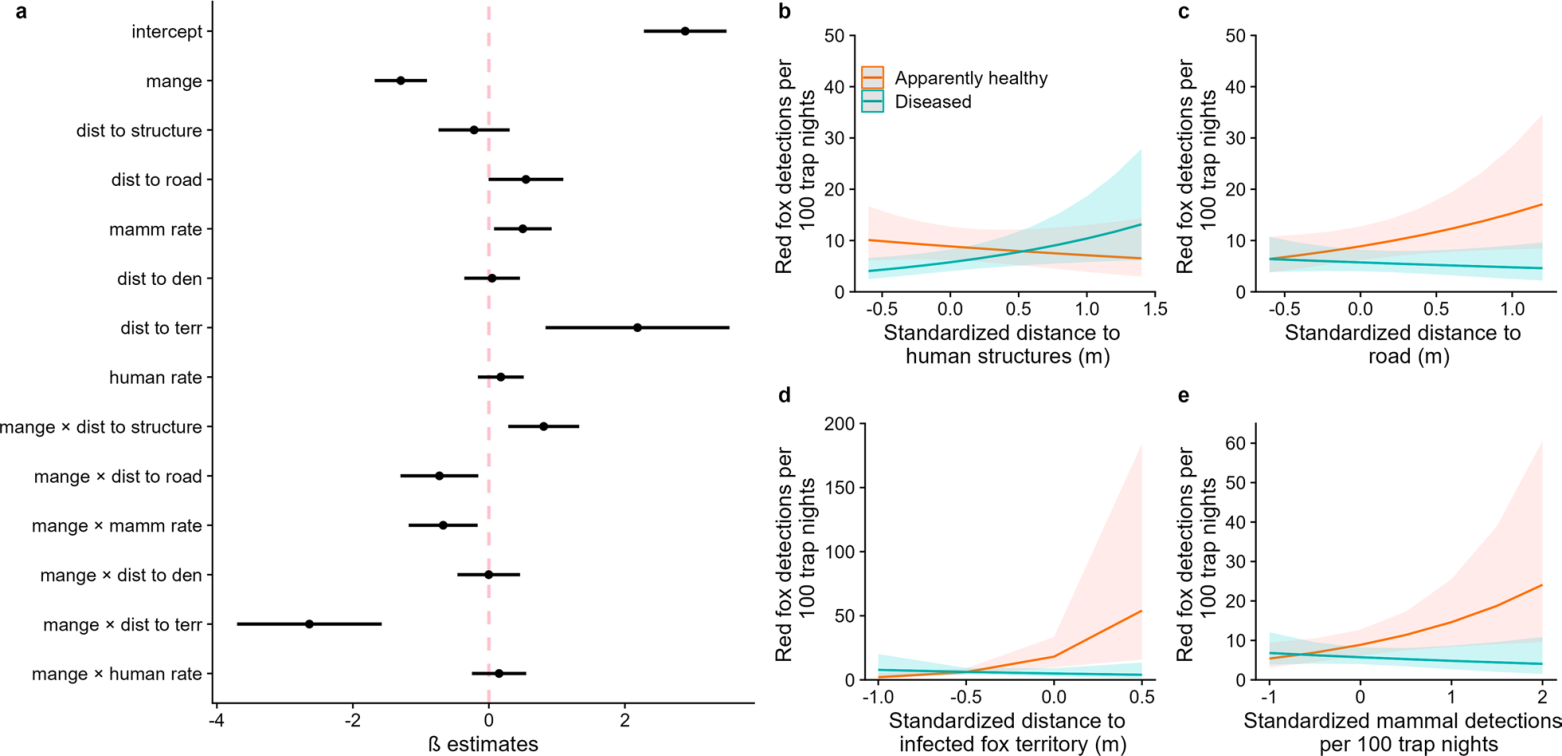
Results of the top-ranked (global) generalized linear mixed-effects model. **a** Beta estimates and 95% confidence intervals (CIs) for covariates included in the top-ranked model. Predicted probability of detecting apparently healthy (orange) and diseased (turquoise) red foxes (*Vulpes vulpes*) in relation to **b** distance to human structures, **c** distance to a paved road, **d** distance to a diseased GPS-collared red fox territory, and **e** detection rates of other mammals. All covariates were standardized by two standard deviations. The solid line illustrates the mean estimate and ribbons depict 95% CIs

The global model was the top-ranked model in our candidate set and received 94.4% of model weight (Appendix 2). Diseased red foxes were more frequently detected away from human structures (Fig. 3a, b) and close to roadways (Fig. 3a, c). Diseased red foxes were also more frequently detected close to the territory of a diseased GPS-collared red fox (Fig. 3a, d) and in areas with frequent detection of other mammals (Fig. 3a, e). We found no evidence that the detection rates of humans and their dogs or distance to previously active red fox dens affected the detection rates of diseased red foxes (Fig. 3a).

### Spatio-temporal spread of sarcoptic mange

**Fig. 4:**
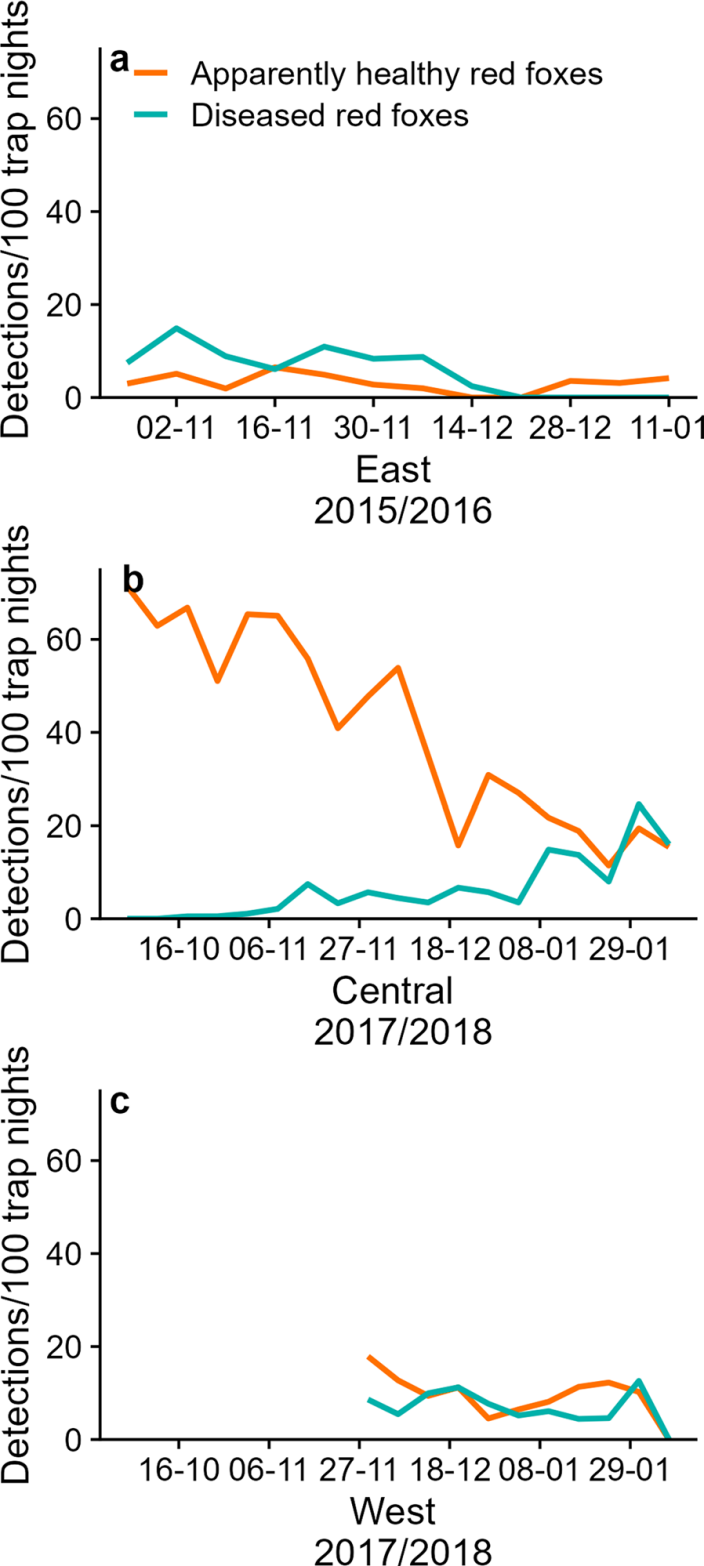
Detection rates (detections per 100 trap nights) of apparently healthy (orange) and diseased (turquoise) red foxes (*Vulpes vulpes*) on Fire Island, NY during the boreal autumn and winters of (**a**) 2015/2016 east of the Old Inlet breach, (**b**) 2017/2018 at central Fire Island, and (**c**) 2017/2018 at the westernmost study area. Dates are listed as day-month

**Fig. 5:**
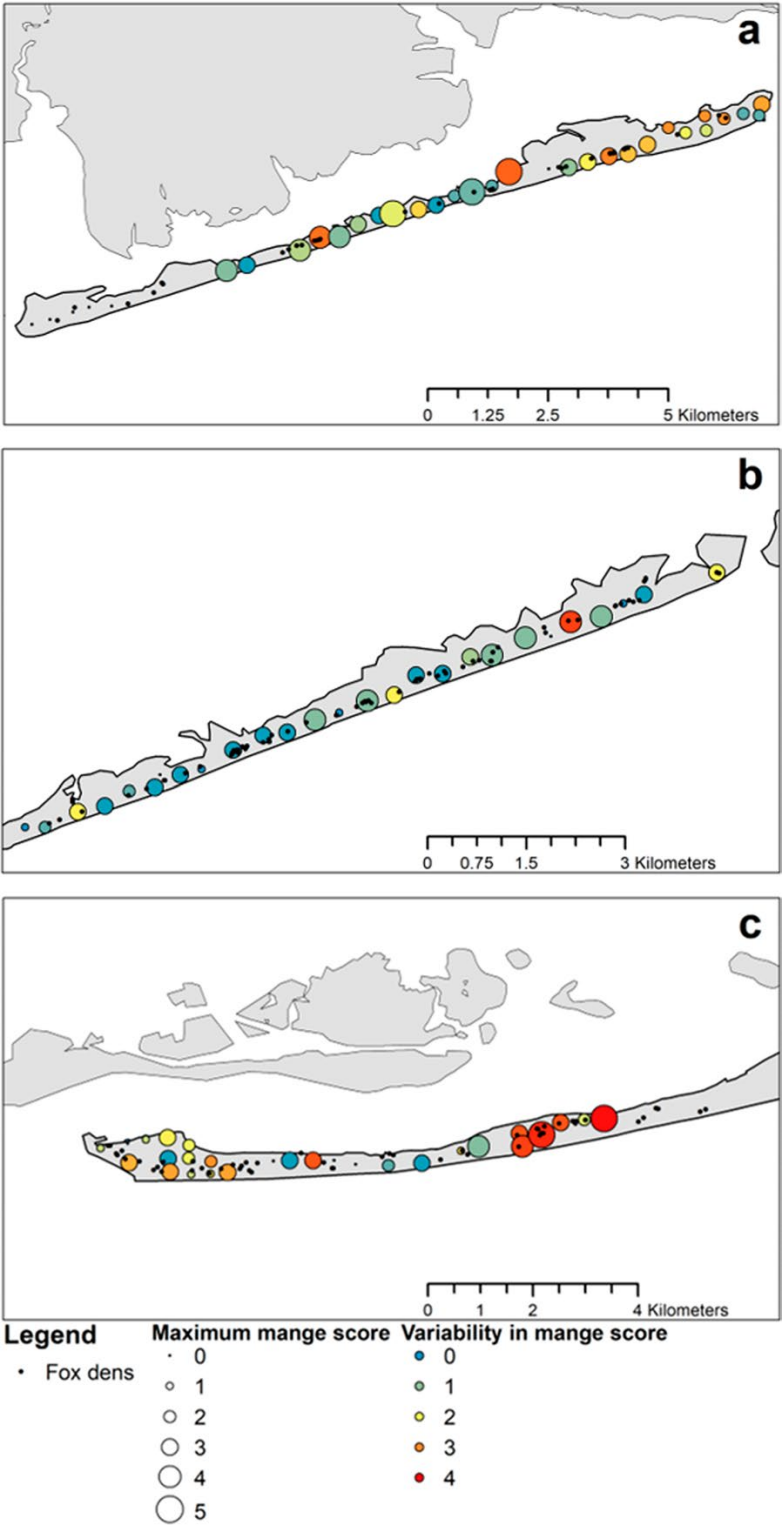
Visual severity of sarcoptic mange in red fox (*Vulpes vulpes*) during the boreal autumn and winters of (**a**) 2015/2016 in the study area east of the Old Inlet breach, (**b**) 2017/2018 at central Fire Island immediately west of the Old Inlet breach, and (**c**) 2017/2018 at the westernmost study area. Black points illustrate locations of fox dens from the previous summer and colored points illustrate locations of camera stations. The size of camera location points reflects the severity (maximum) and color reflects variability (interquartile range)

The first outbreak of sarcoptic mange observed east of the Old Inlet breach coincided with our 2015/2016 camera trapping session (26 October 2015–4 February 2016). Red fox detections in this area were low (276 red fox detections, 15.8 red fox detections/100 trap nights, 12.0% of all animal detections), and 70.4% of those detections were of diseased individuals (Fig. 4a). The area where diseased red foxes were photographed increased slowly from 63.8 ha (9.8% of the monitored area), peaked 3 weeks later at 85.7 ha (spreading approximately 7.3 ha per week to 13.2% of the monitored area, a 34.3% increase), then declined to 60.9 ha (9.3% of the monitored area). The median visual severity of diseased red foxes was greatest in the interior of the eastern segment of Fire Island (Fig. 5a) and was fairly stable throughout the camera trapping session (median ± interquartile range = 3 ± 1, max = 5).

The second sarcoptic mange outbreak at the western portion of Fire Island overlapped with the 2017/2018 camera trapping session (29 September 2017–16 March 2018). Red fox detections during the second outbreak were high relative to the outbreak that occurred east of the Old Inlet breach (2302 red fox detections, 43.0 red fox detections/100 trap nights, 19.9% of all animal detections) and only 19.0% of red fox detections were of diseased individuals (Fig. 4b, c). The second sarcoptic mange outbreak appeared to spread from west to east. At the westernmost end of Fire Island, the visual severity of diseased red foxes increased throughout the camera trapping session (median ± interquartile range late November = 2 ± 1, median ± interquartile range late January = 3 ± 0; max = 5), and the area where diseased red foxes were photographed peaked at 212.2 ha (51.2% of the monitored area) in late December, with a gradual decrease to 87.3 ha over the next 2 weeks (21.3% of the monitored area, a 58.9% decrease). In the center of Fire Island immediately west of the Old Inlet breach, the visual severity of diseased red foxes was lowest of all three study areas (median ± interquartile range = 0 ± 0, max = 4), and the area where diseased red foxes were photographed increased from 0.3 ha (< 0.1% of the monitored area) to 96.8 ha 8 weeks later (spreading approximately 12.1 ha per week to 20.5% of the monitored area and a > 300-fold increase). The severity and variability of sarcoptic mange during the second outbreak were greatest around human infrastructure (Fig. 5b, c).

## Discussion

The sarcoptic mange outbreak in the red fox population on Fire Island led to the temporary extirpation of red foxes on the smaller, eastern portion of the island and reduced abundance of red foxes on the larger, western portion. No other skin disease with comparable visible signs (e.g., fungal infections) was observed or diagnosed in red foxes at the time of our study, and our molecular methods revealed high similarity to known *S. scabiei* sequences. Thus, our study reflects a conservative evaluation for the spread of sarcoptic mange. Based on the severity of disease and frequency of red fox detections, we suspect that we captured the initial outbreak of sarcoptic mange in central Fire Island, mid-stage spread at the westernmost area of Fire Island, and the late stage of the disease and subsequent subpopulation extirpation east of the Old Inlet breach.

Because sarcoptic mange does not spread linearly across a population or ecosystem, tracking the prevalence of disease does not necessarily yield obvious patterns [53]. Moreover, individuals infected with *S. scabiei* mites may not present visible signs of the mange disease for several weeks post-exposure, further obscuring patterns of sarcoptic mange spread [3, 25, 44]. Our analyses suggest that sarcoptic mange transmission was driven by red fox movement, in line with Devenish-Nelson et al. [54], who found no evidence for environmental transmission in red foxes. We detected diseased red foxes more frequently when close to locations of other diseased GPS-collared red foxes. *Sarcoptes scabiei* mites likely spread between red foxes, perhaps by a few transient individuals [28].

Red foxes with advanced stages of sarcoptic mange were photographed closer to developed areas, likely because diseased red foxes increased their reliance on anthropogenic subsidies [24, 55]. Those same red foxes with advanced stages of sarcoptic mange likely succumbed to the disease, possibly explaining the apparent negative relationship between detection rates of diseased red foxes and distance to human structures. Additionally, our camera trapping study occurred during the boreal autumn and winter, when there are fewer beach recreationists relative to boreal summer months and only certain parts of the park are accessible, resulting in fewer areas across the landscape where red foxes may find anthropogenic subsidies. In response to seasonal changes in anthropogenic subsidies, diseased red foxes may have used roadways for travel and remained close to open park facilities as their disease worsened.

It remains unknown exactly how *S. scabiei* mites arrived at Fire Island, and our current understanding of *S. scabiei* genetic structure, combined with their apparent slow evolution, may be insufficient for high-resolution host differentiation [10, 56]. Although the ITS-2 sequence has been demonstrated to be genetically polymorphic [33, 57], we found a single nucleotide variation at position 362 when our sequence was compared with the *S. scabiei* isolate Fox23c from northern Italy [57]. The *S. scabiei* sequence we obtained exhibited a nucleotide substitution at position 298 (T instead of an A) that allows us to genetically discriminate *S. scabiei* mites collected in red foxes from those collected from chamois (*Rupicapra* sp.) [57], though any further inference is limited. It is possible that *S. scabiei* mite lineages could be revealed using whole-genome sequencing [10].

The spatio-temporal nature of the two sarcoptic mange outbreaks we observed separated by > 40 km and over a year suggests that *S. scabiei* mites could have been transferred to Fire Island during two separate emigration events from Long Island. The two red fox subpopulations we monitored were isolated by the strong currents of the Old Inlet breach [28], though each section of Fire Island is accessible from Long Island by bridge and when sections of the Great South Bay freeze during the winter. We frequently detected white-tailed deer, feral cats, and northern raccoons across the study area, which were an important predictor in our analyses. Each of these species is a potential sarcoptic mange vector ( [41–43], but see [2]) that could transfer *S. scabiei* mites from Long Island, and clinical signs of the disease may not be visible [11, 41].

In continental systems, sarcoptic mange epizootics can occur every 20–40 years and are typically followed by a longer c. 50-year enzootic phase as the red fox population develops immunity [8, 15]. While it is possible that sarcoptic mange existed in an enzootic phase around the interior villages at Fire Island, this would not explain the epizootic at the eastern study area. East of Old Inlet, red foxes were extirpated by March 2016, with intermittent presence of a few red foxes believed to use a bridge that connects Fire Island to Long Island. It may be that the Fire Island red fox population is part of a larger metapopulation with red foxes on Long Island, where sarcoptic mange could exist in an enzootic phase. Transient red foxes from Long Island, like those believed to visit the east study area in 2017 and 2018, could potentially transfer *S. scabiei* mites to the relatively “naïve” Fire Island red fox subpopulation. Regardless of how *S. scabiei* mites arrived on Fire Island, outbreaks of sarcoptic mange are likely an important driver of red fox population dynamics in this system.

## Conclusions

Monitoring for sarcoptic mange outbreaks among red fox is important for land managers, as the efficacy of lethal predator control (e.g., to protect co-occurring threatened and endangered species) could vary with the red fox population size [58]. Remote cameras are a valuable, noninvasive tool to detect and monitor outbreaks of sarcoptic mange in red fox populations (e.g., [18, 41]). However, we caution against the sole use of remote cameras to diagnose suspected sarcoptic mange outbreaks without concomitant diagnostic sampling (e.g., mite identification from skin scrapings [2], molecular methods [32]) as visible signs can be misidentified [41]. Once verified through laboratory diagnostics, the degree of visual signs can be observed with remote cameras to monitor the severity and spread of the mange disease. Land managers should be aware of and understand the parasitology of sarcoptic mange and barrier island ecology to observe the spatio-temporal spread and progress of clusters around human development for public health and safety (e.g., [8]). Future research quantifying population responses to sarcoptic mange epizootics may be able to use unique patterns of alopecia and hyperkeratosis, similar to pelage patterns that are used to identify individuals of other wildlife taxa (e.g., [59]), to identify individuals to better understand movement and activity patterns.

### Supplementary Information

Supplementary Material. 1

## Data Availability

The data that support the findings of this study are available from the corresponding author upon reasonable request. Sequences have been deposited in the NCBI database under accession numbers PP795437 and PP795438.
