# Supplementary material for: Epidemiology of sarcoptic mange in a geographically constrained insular red fox population
Source: Parasit Vectors. 2024 Jun 6;17:248. doi: 10.1186/s13071-024-06330-5 (PMC11157703; doi:10.1186/s13071-024-06330-5)
Supplement: Supplementary file 1 — Supplementary Material. 1 [file 13071_2024_6330_MOESM1_ESM.docx]

Appendix 1. Visual severity of sarcoptic mange signs and corresponding rank.

| **Rank** | | **Description of visible signs** | **Example** |
| --- | --- | --- | --- |
| Apparently healthy | 0 | No signs of visible mange. An apparently healthy individual or an individual that is not yet exhibiting signs. | 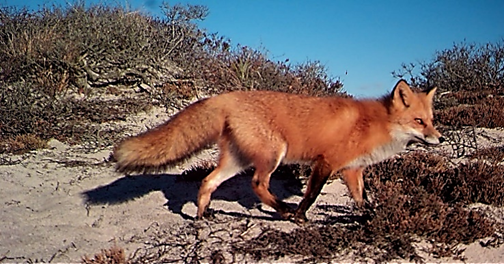 |
| Diseased | 1 | Mild signs of mange, mostly limited to hyperkeratosis or alopecia of the face.^a^ | 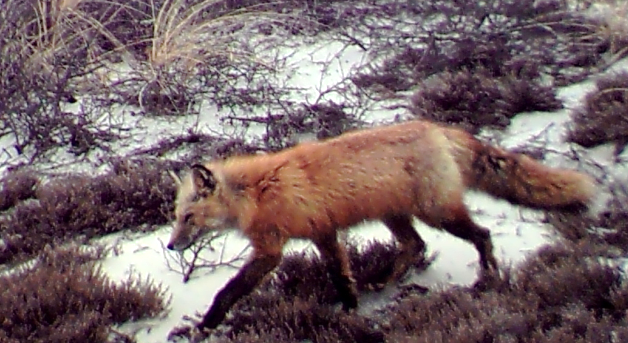 |
|  | 2 | Mange spreading. Hyperkeratosis or alopecia on face and spreading to legs (and ≤ 5% of visible torso).^a^ | 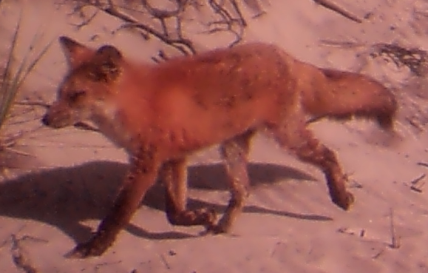 |
|  | 3 | Moderate disease. Hyperkeratosis, alopecia, or exposed gray skin spreading to tail (and ≤ 15% of visible torso).^b^ | 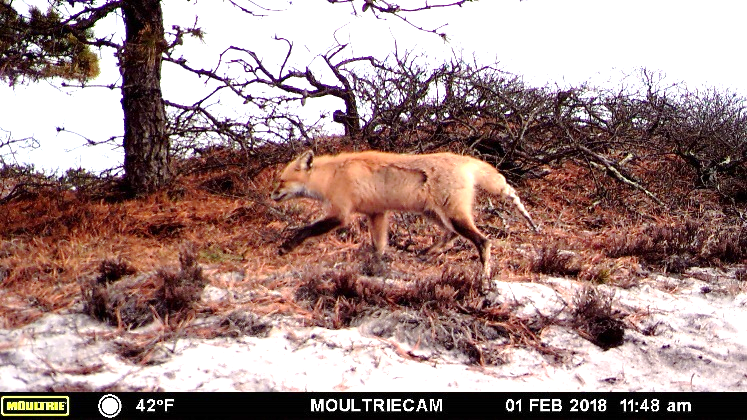 |
|  | 4 | Worsening disease. Hyperkeratosis, alopecia, or exposed gray skin on c. 50% of visible body surface.^b^ | 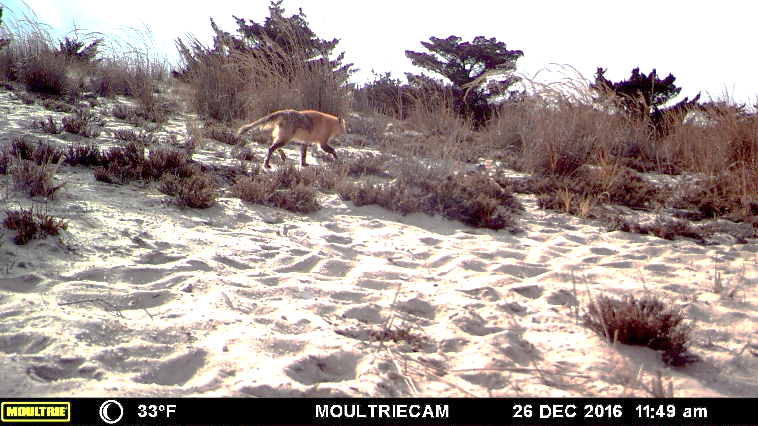 |
|  | 5 | Severe disease. Visible mange signs on ≥ 50% of visible body surface.^c^ | 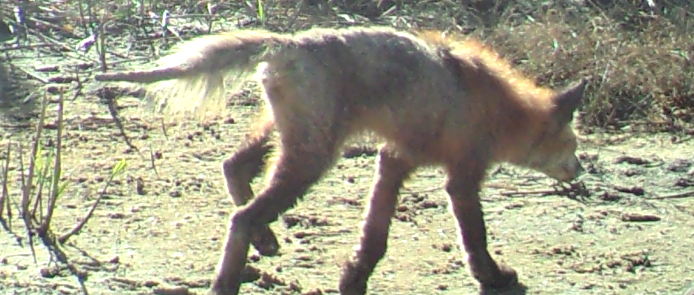 |

^a^ Approximates Class I [13]: small infections (c. 1 cm2) of fore and hind limbs, ischium, and ears.

b Approximates Class II [13]: increased infection size (≤ 50% of body surface), knees and elbows.

c Approximates Class III [13]: infection size across most of the body (> 50% of body surface).

Appendix 2. Generalized linear mixed-effect models (negative binomial error distribution) ranked by Akaike’s information criterion corrected for sample size (AIC*_c_*) evaluating detections of red foxes (*Vulpes vulpes*). The covariate ‘mange’ (0/1) was included in all models, including the null. The global model contained all two-way interactions from other candidate models and the constituent (additive) terms. Number of parameters (*k*), log likelihood, difference in AIC*_c_* score between the top-ranked model and model *i* (ΔAIC*_c_*), and model weight (ω*_i_*) are shown.

| **Model** | ***k*** | **Log likelihood** | **AIC*_c_*** | **ΔAIC*_c_*** | **ω*_i_*** |
| --- | --- | --- | --- | --- | --- |
| Global | 17 | -552.43 | 1142.22 | 0.00 | 0.94 |
| Dist to terr + mange + dist to terr × mange | 7 | -566.65 | 1147.88 | 5.66 | 0.06 |
| Dist to road + mange + dist to road × mange | 7 | -575.97 | 1166.52 | 24.31 | < 0.01 |
| Human rate + mange + human rate × mange | 7 | -589.85 | 1194.29 | 52.08 | < 0.01 |
| Dist to structure + mange + dist to structure × mange | 7 | -590.31 | 1195.20 | 52.98 | < 0.01 |
| Null | 5 | -593.53 | 1197.38 | 55.16 | < 0.01 |
| Mamm rate + mange + mamm rate × mange | 7 | -591.88 | 1198.34 | 56.13 | < 0.01 |
| Dist to den + mange + dist to den × mange | 7 | -593.12 | 1200.82 | 58.61 | < 0.01 |

Dist to structure: distance to nearest human structure

Dist to road: distance to nearest paved road

Dist to den: distance to nearest active den from the previous boreal summer

Dist to terr: distance to nearest territory of a diseased GPS-collared red fox

Mamm rate: combined detection rate of feral cats, white-tailed deer, and northern raccoons

Human rate: detection rates of humans and their dogs

Mange: visible sign of sarcoptic mange
